# Supplementary material for: Stochastic gene expression in auxin signaling in the floral meristem of Arabidopsis thaliana
Source: Nat Commun. 2025 May 20;16:4682. doi: 10.1038/s41467-025-59943-4 (PMC12092670; doi:10.1038/s41467-025-59943-4)
Supplement: Supplementary file 1 — Supplementary Information [file 41467_2025_59943_MOESM1_ESM.pdf]

Stochastic gene expression in auxin signaling in the floral meristem of *Arabidopsis thaliana*

Kong *et al.*

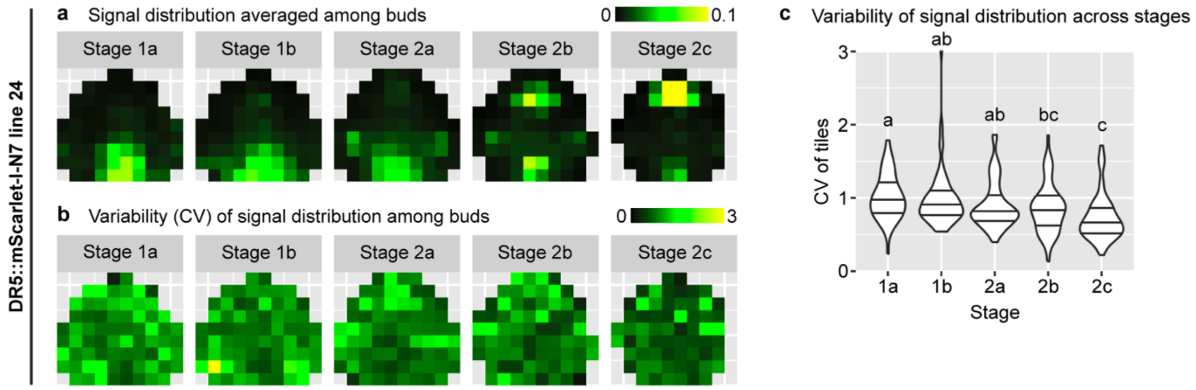

Supplementary Fig. 1. Analysis of an independent insertion line of *DR5::mScarlet-I-N7*. Mean (**a**) and variability (CV) (**b-c**) of signal distribution patterns calculated from these buds: stage 1a, n=16; stage 1b, n=15; stage 2a, n=21; stage 2b, n=10; stage 2c, n=6. In (**c**), each data point is a CV value at an (x,y) tile of a given stage. Lines show quartiles. Letters denote statistical significance in pairwise two-sided permutation tests with Bonferroni's *p*-value adjustments. Adjusted *p*-values: 1a-1b, 1.000, 1a-2a, 0.403, 1a-2b, 0.046, 1a-2c, 0.000, 1b-2a, 1.000, 1b-2b, 0.387, 1b-2c, 0.000, 2a-2b, 1.000, 2a-2c, 0.012, 2b-2c, 0.213. Note that variability of global DR5 pattern decreases with developmental stage. Source data are provided as a Source Data file.

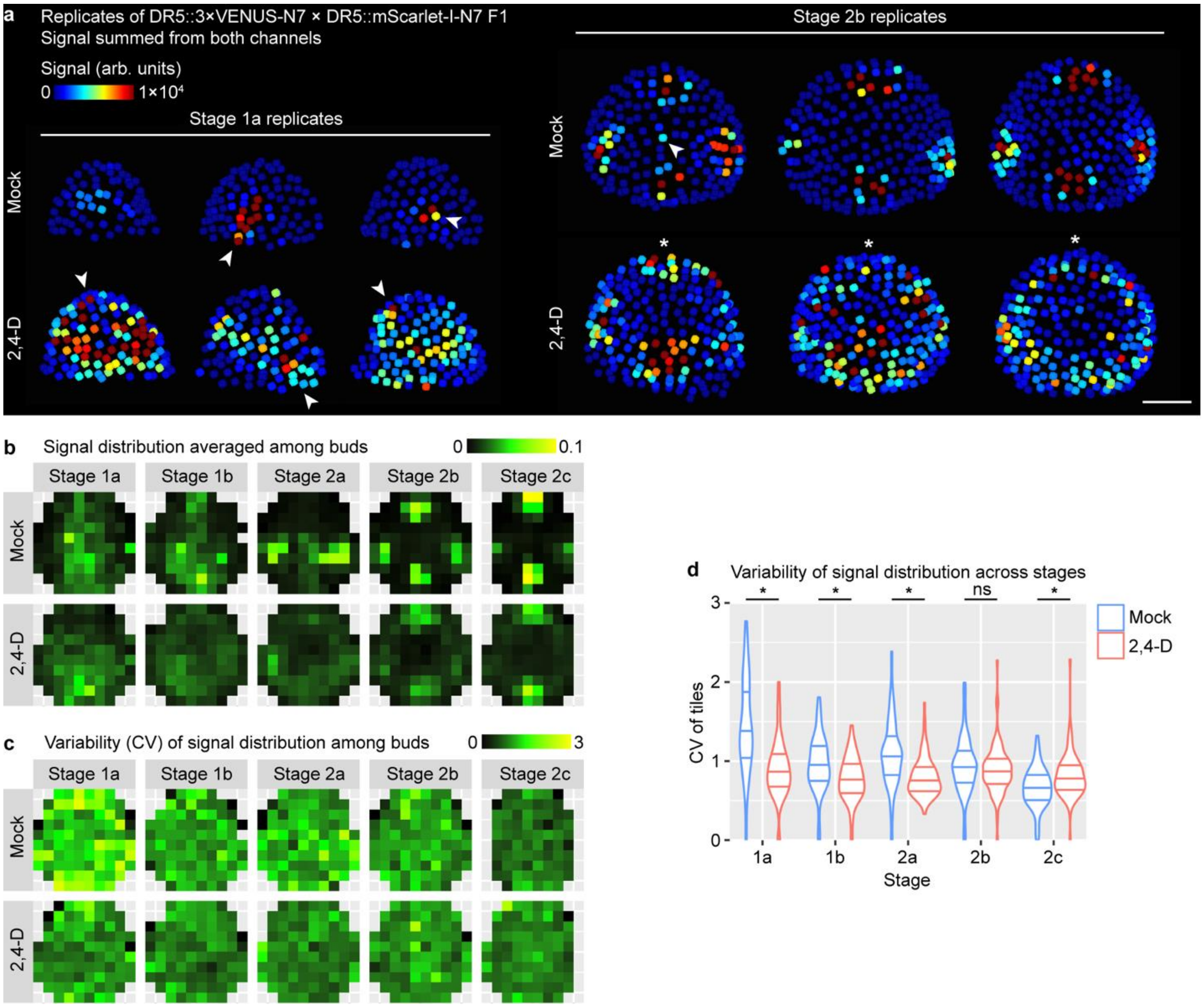

Supplementary Fig. 2. Variability in DR5 signal persists in high auxin levels. a Three replicates of DR5::3×VENUS-N7 × DR5::mScarlet-I-N7 F1 meristems treated with mock or 2,4-D, showing summed signal from both channels. Arrowheads, sporadic patches of high auxin signaling. Asterisks, even in 2,4-D treated meristems, the same position has different levels of auxin signaling across replicates. Scale bar, 20 µm. **b-d** Mean (**b**) and variability (CV) (**c-d**) of signal distribution patterns. Stage 1a, n=18 buds per treatment. Stage 1b, n=10 for mock and n=13 for 2,4-D. Stage 2a, n=10 for mock and n=12 for 2,4-D. Stage 2b, n=9 for mock and n=10 for 2,4-D. Stage 2c, n=3 for mock and n=10 for 2,4-D. In (**d**), each data point is a CV value at an (x,y) tile of a given stage; asterisks show statistical significance from two-sided permutation tests with Bonferroni's *p*-value adjustments. Adjusted *p*-values: 1a, 0, 1b, 6.5×10^{-4}, 2a, 0, 2b, 1, 2c, 2.2×10^{-3}. Source data are provided as a Source Data file.

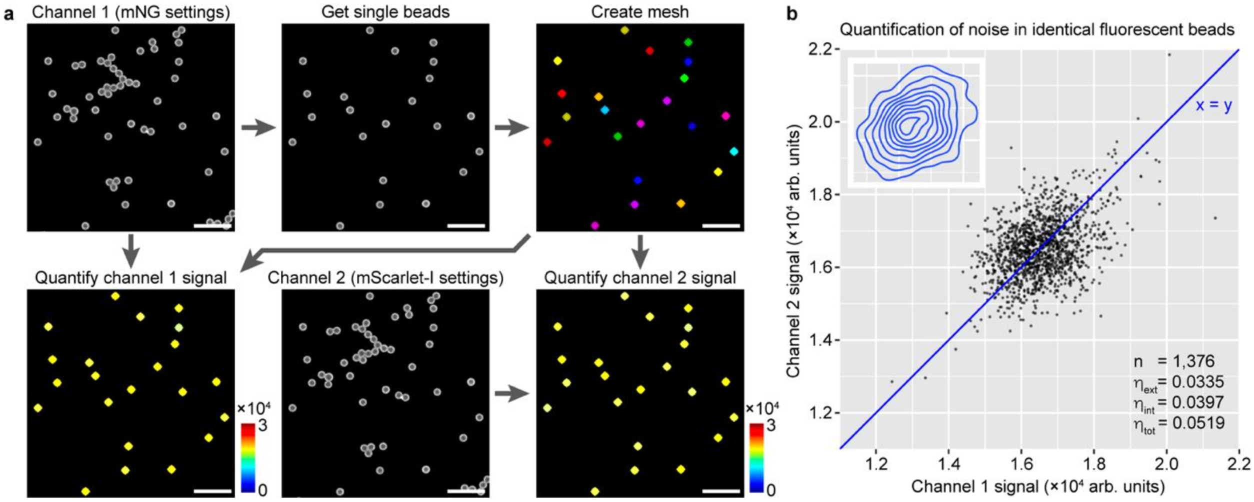

Supplementary Fig. 3. Quantification of extrinsic and intrinsic noise caused by instrument and measurement errors. a Experimental procedure. Fluorescent beads were imaged using mNG and mScarlet-I settings. Single beads were used to create meshes, which were then used to quantify signal from both channels. Scale bars, 20 μm. **b** Dot plot and quantification of extrinsic (η_{ext}), intrinsic (η_{int}), and total noise (η_{tot}) from n=1,376 beads. Inset shows the distribution of dots visualized by a 2D density plot. Source data are provided as a Source Data file.

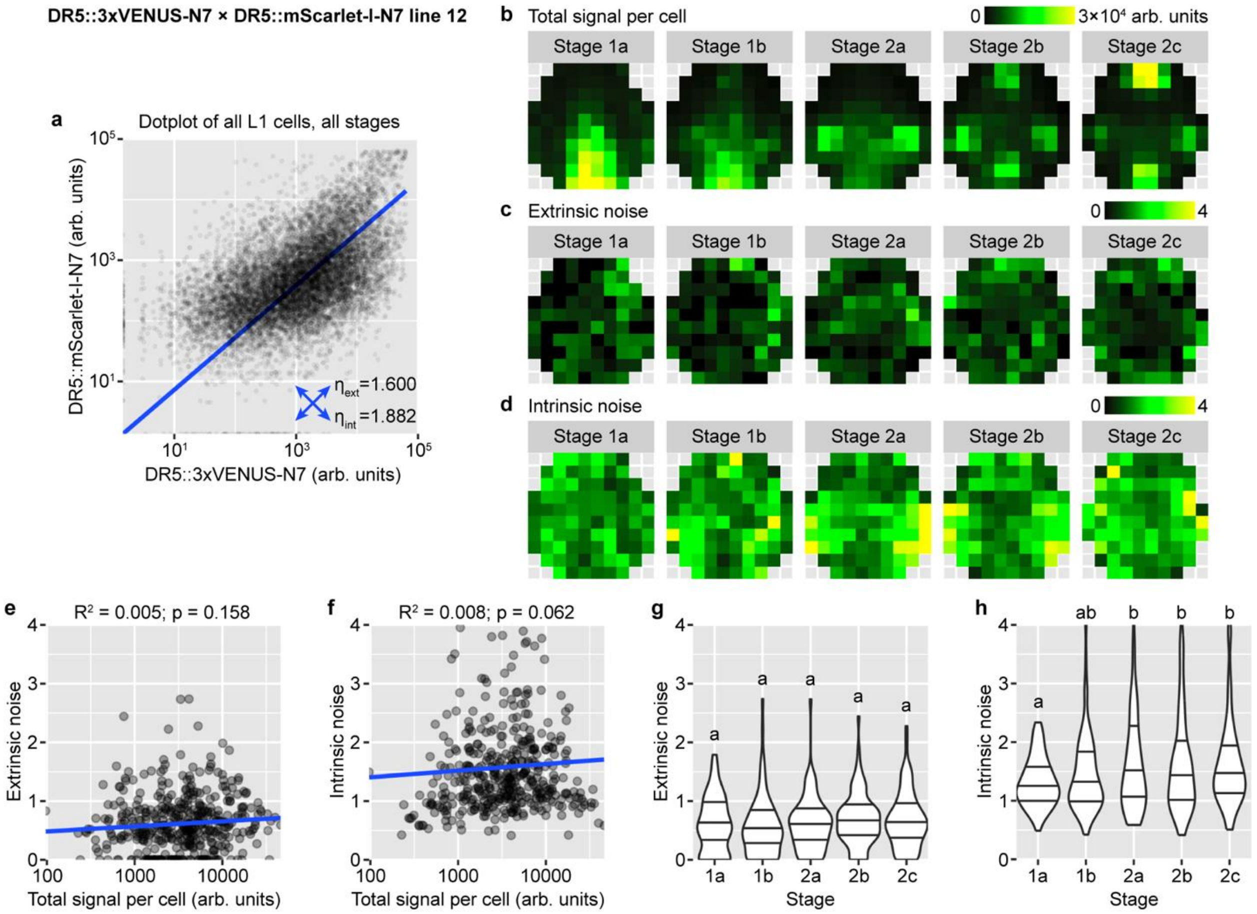

Supplementary Fig. 4. Analysis of an independent dual reporter line of DR5. a Dot plot of dual-reporter signals of all cells from buds of all stages. Number of buds: stage 1a, n=23; stage 1b, n=16; stage 2a, n=13; stage 2b, n=10; stage 2c, n=8. **b** Summed signal from both channels, averaged across all cells in each tile. **c-d** Extrinsic (**c**) and intrinsic (**d**) noise calculated from all cells in each tile, which lack apparent spatial patterns. **e-h** Relation of extrinsic and intrinsic noise to total signal per cell (**e-f**) and developmental stage (**g-h**). Each data point is a noise value at an (x,y) tile of a given stage. In (**g-h**), lines show quartiles; letters denote statistical significance in pairwise two-sided permutation tests with Bonferroni's *p*-value adjustments. Adjusted *p*-values for (**g**): 1b-2b, 0.681, others, 1.000. Adjusted *p*-values for (**h**): 1a-1b, 0.392, 1a-2a, 0.002, 1a-2b, 0.028, 1a-2c, 0.007, others, 1.000. In contrast to variability in global DR5 pattern across buds which decreases from stage 1 to 2, cellular noise either does not change (extrinsic noise) or slightly increases (intrinsic noise) with developmental stage. Source data are provided as a Source Data file.

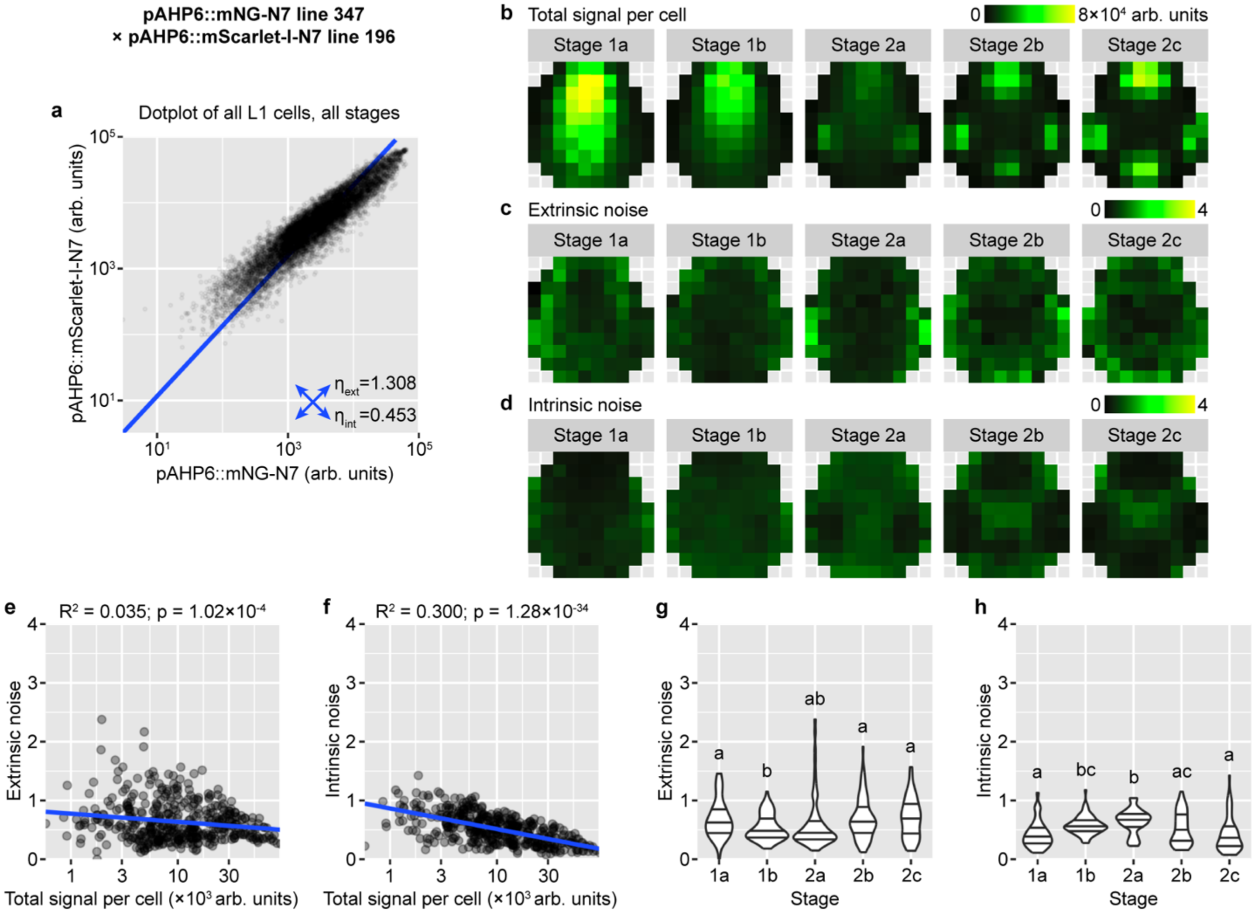

Supplementary Fig. 5. Analysis of an independent dual reporter line of *AHP6*. a Dot plot of dual-reporter signals of all cells from buds of all stages. Number of buds: stage 1a, n=14; stage 1b, n=14; stage 2a, n=12; stage 2b, n=10; stage 2c, n=6. **b** Summed signal from both channels, averaged across all cells in each tile. **c-d** Extrinsic (**c**) and intrinsic (**d**) noise calculated from all cells in each tile. Note that extrinsic noise is higher in the peripheral zone than in the central zone; intrinsic noise is lower in the incipient sepals than non-sepal regions. **e-f** Extrinsic and intrinsic noise are negatively correlated with total signal per cell. **g-h** Relation of extrinsic and intrinsic noise to developmental stage. Note that stages 1b and 2a has low extrinsic noise and high intrinsic noise than the rest of the stages. In (**e-h**), each data point is a noise value at an (x,y) tile of a given stage. In (**g-h**), lines show quartiles; letters denote statistical significance in pairwise two-sided permutation tests with Bonferroni's *p*-value adjustments. Adjusted *p*-values for (**g**): 1a-1b, 0.004, 1a-2a, 0.843, 1a-2b, 1.000, 1a-2c, 1.000, 1b-2a, 1.000, 1b-2b, 0.004, 1b-2c, 0.003, 2a-2b, 0.825, 2a-2c, 0.694, 2b-2c, 1.000. Adjusted *p*-values for (**h**): 1a-1b, 0.000, 1a-2a, 0.000, 1a-2b, 0.127, 1a-2c, 1.000, 1b-2a, 0.129, 1b-2b, 1.000, 1b-2c, 0.000, 2a-2b, 0.017, 2a-2c, 0.000, 2b-2c, 0.144. Source data are provided as a Source Data file.

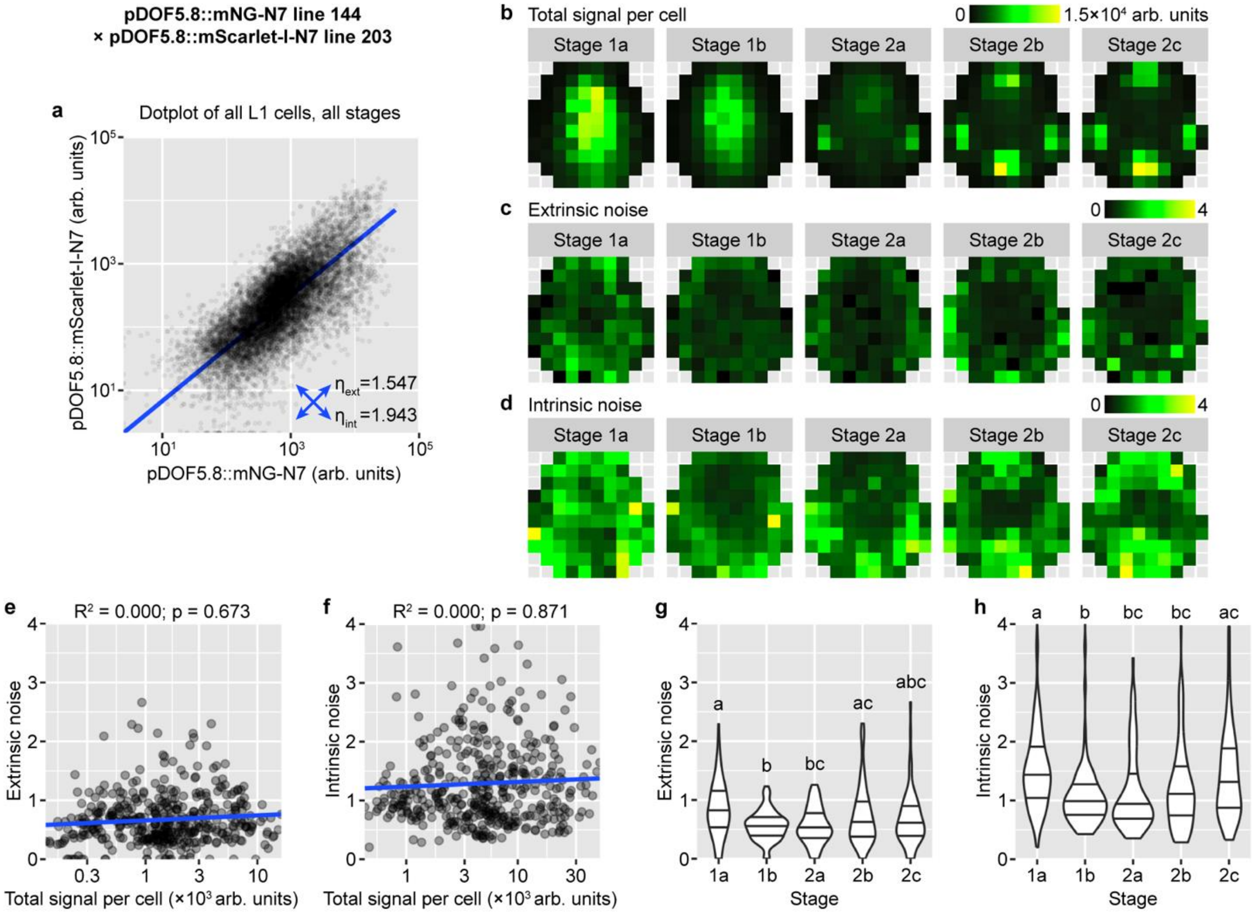

Supplementary Fig. 6. Analysis of an independent dual reporter line of *DOF5.8*. a Dot plot of dual-reporter signals of all cells from buds of all stages. Number of buds: stage 1a, n=13; stage 1b, n=17; stage 2a, n=10; stage 2b, n=10; stage 2c, n=10. **b** Summed signal from both channels, averaged across all cells in each tile. **c-d** Extrinsic (**c**) and intrinsic (**d**) noise calculated from all cells in each tile. Note that extrinsic noise is higher in the peripheral zone than in the central zone; intrinsic noise is higher in the incipient sepals than non-sepal regions. **e-f** Extrinsic and intrinsic noise are not correlated with total signal per cell. **g-h** Relation of extrinsic and intrinsic noise to developmental stage. Note that extrinsic and intrinsic noise decrease from stage 1a to 2a and then increase in stage 2b and 2c. In (**e-h**), each data point is a noise value at an (x,y) tile of a given stage. In (**g-h**), lines show quartiles; letters denote statistical significance in pairwise two-sided permutation tests with Bonferroni's *p*-value adjustments. Adjusted *p*-values for (**g**): 1a-1b, 0.000, 1a-2a, 0.000, 1a-2b, 1.000, 1a-2c, 0.243, 1b-2a, 1.000, 1b-2b, 0.046, 1b-2c, 0.568, 2a-2b, 0.084, 2a-2c, 0.924, 2b-2c, 1.000. Adjusted *p*-values for (**h**): 1a-1b, 0.000, 1a-2a, 0.001, 1a-2b, 0.020, 1a-2c, 1.000, 1b-2a, 1.000, 1b-2b, 1.000, 1b-2c, 0.013, 2a-2b, 1.000, 2a-2c, 0.111, 2b-2c, 0.715. Source data are provided as a Source Data file.

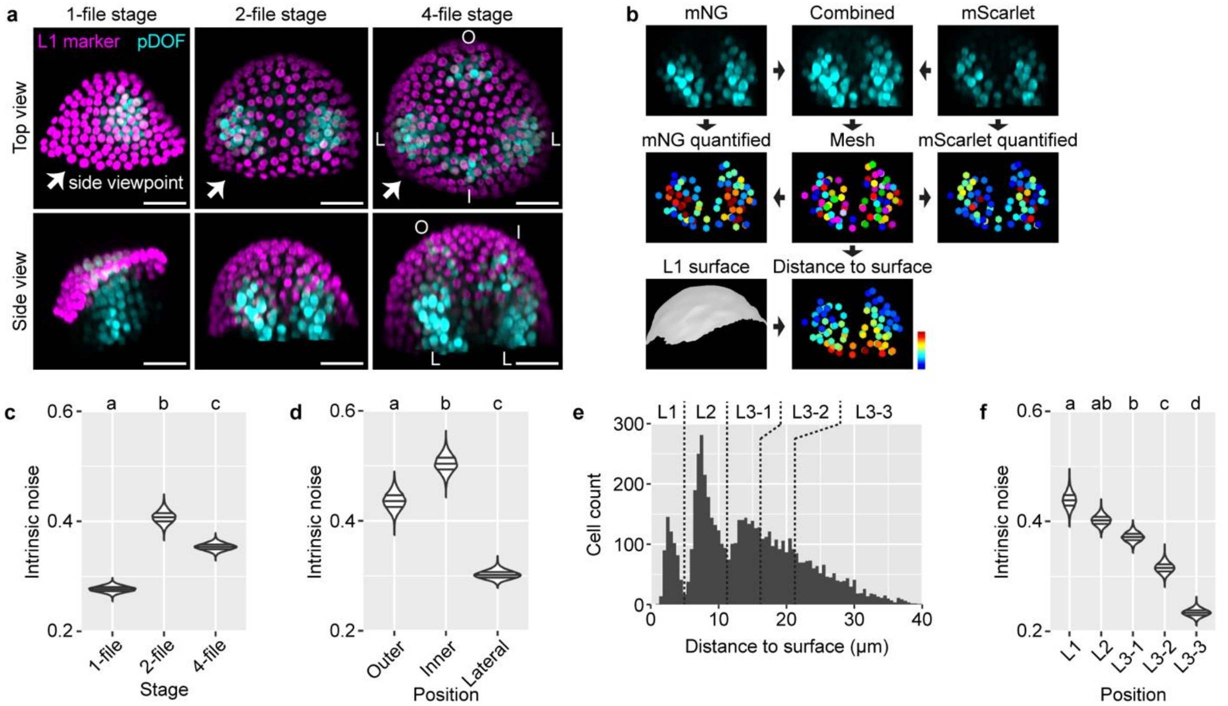

Supplementary Fig. 7. Analysis of the *DOF5.8* dual reporter in all cell layers reveals positional dependency of intrinsic noise. a Representative images of *DOF5.8* expression pattern across stages. Magenta, *pATML1::H2B-TFP*. Cyan, combined signal from *pDOF5.8::mNG-N7* and *pDOF5.8::mScarlet-I-N7*. 1-file stage roughly corresponds to stage 1a to 1b; 2-file stage roughly corresponds to stage 1b to 2a; 4-file stage roughly corresponds to stage 2a to 2c. In 4-file stage, letters denote *DOF5.8*-expressing cell files in the incipient outer (O), inner (I), and lateral (L) sepal regions. Scale bars, 20 µm. **b** Workflow for quantifying the *DOF5.8* dual reporter in all cell layers. Signal from both *DOF5.8* reporters were combined, and nuclei with signal from either reporter were used to create the nuclear mesh. The nuclear mesh was used to quantify signal from either channel, with which intrinsic noise in gene expression was calculated. Distance of each nucleus to the bud surface was calculated using a surface mesh made from the epidermal marker *pATML1::H2B-TFP*. **c** Intrinsic noise is highest in the 2-file stage. **d** For buds in the 4-file stage, intrinsic noise is higher in the incipient outer and inner sepals than the incipient lateral sepals. **e** Histogram of distances from nuclei to the bud surface. Nuclei were divided into L1 (0-5 µm), L2 (5-11 µm), and artificially, L3-1 (11-16 µm), L3-2 (16-21 µm), and L3-3 (>21 µm). **f** Intrinsic noise decreases with depth in the tissue. Number of buds quantified: 1-file stage, n=25; 2-file stage, n=12; 4-file stage, n=18. In (**c**, **d**, **f**), violin plots show the distribution of 10,000 bootstrapped samples, and lines show quartiles. Letters indicate statistical significance in two-sided permutation tests with Bonferroni's *p*-value adjustment. Adjusted *p*-values for (**c**): 1-2, 0, 1-4, 0, 2-4, 1.083×10^{-2}. Adjusted *p*-values for (**d**): 0 for each pairwise comparison. Adjusted *p*-values for (**f**): L1-L2, 1.000, L1-L3-1, 0.035, L1-L3-2, 0.000, L1-L3-3, 0.000, L2-L3-1, 0.933, L2-L3-2, 0.000, L2-L3-3, 0.000, L3-1-L3-2, 0.025, L3-1-L3-3, 0.000, L3-2-L3-3, 0.000. Source data are provided as a Source Data file.

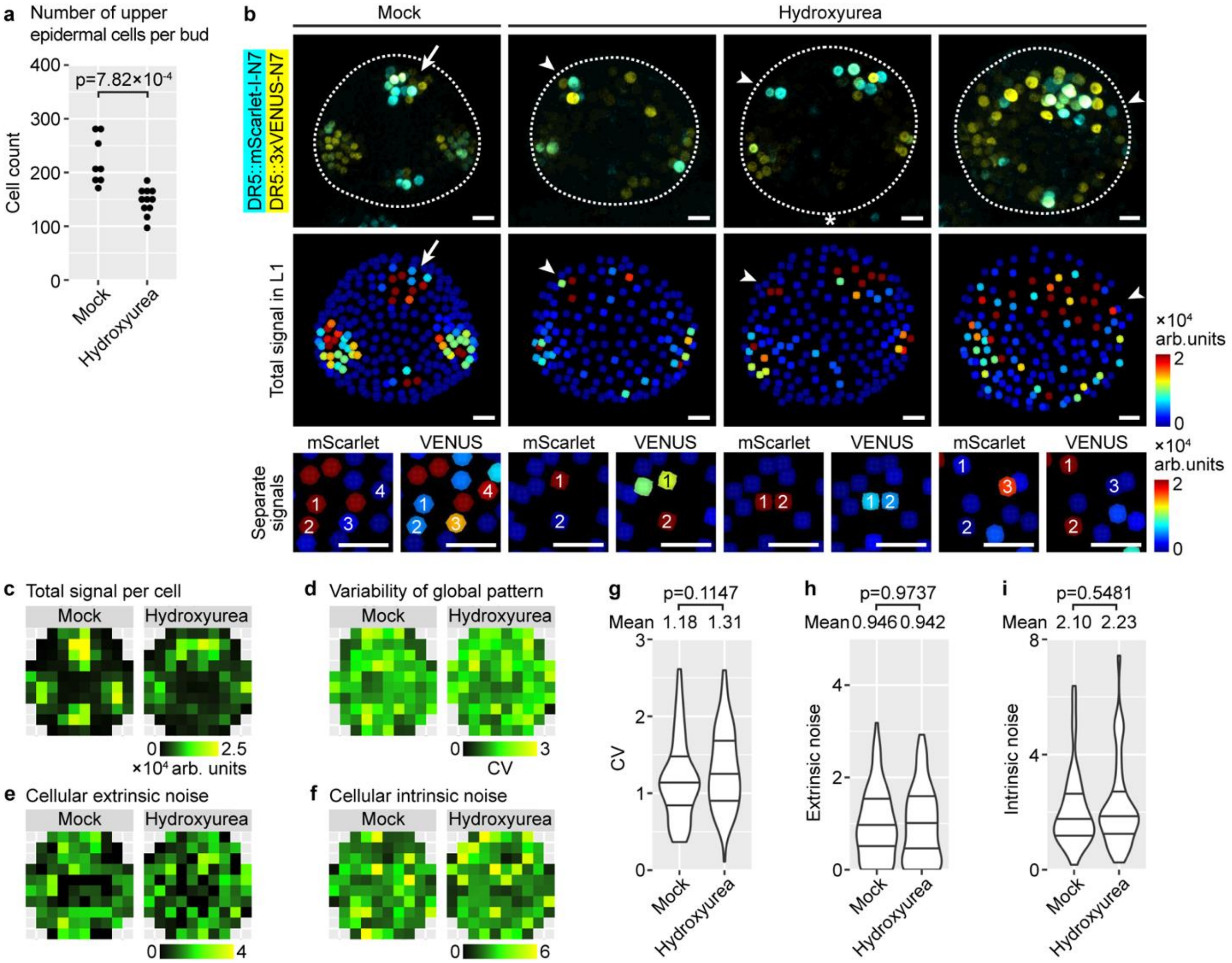

Supplementary Fig. 8. Hydroxyurea treatment subtly increases variability of global pattern. a Hydroxyurea treatment decreases cell number in the upper epidermis of stage 2 meristems. To ensure matching of developmental stage, the oldest stage 2 buds from each inflorescence were analyzed. Mock, n=8 buds. Hydroxyurea, n=11 buds. *p*-value, two-sided Wilcoxon's rank sum test. **b** Stage 2 meristems of DR5 dual reporter treated with mock or hydroxyurea. Top, overlaid signal from both reporters. Middle, summed signal from both DR5 reporters in L1, showing global pattern of auxin signaling. Arrow, an incipient sepal primordium in which not all cells express both copies of DR5 but still makes an auxin signaling maxima. Asterisk, a region where an inner sepal primordium normally initiates; however, this region has no DR5-positive cells and does not make an auxin signaling maximum. Arrowheads, cells outside regions where sepal primordia normally initiate but expressing DR5 stochastically, making exogenous auxin signaling maxima. Bottom, separate mScarlet and VENUS signals, showing both mock-treated and hydroxyurea-treated buds were impacted by intrinsic noise. Scale bars, 10 µm. **c** Summed signal from both channels, averaged across all cells in each tile. **d** Variability of global DR5 pattern across samples. **e** Cellular extrinsic noise. **f** Cellular intrinsic noise. **g** Variability of global DR5 pattern slightly yet non-significantly increases upon hydroxyurea treatment. **h**-**i** Cellular extrinsic noise (**h**) and intrinsic noise (**i**) are unchanged by hydroxyurea. For (**g**-**i**), each data point is a value of CV, extrinsic noise, and intrinsic noise, respectively, calculated from an (x,y) tile. Lines show quartiles. *p*-values are from two-sided permutation tests. Source data are provided as a Source Data file.

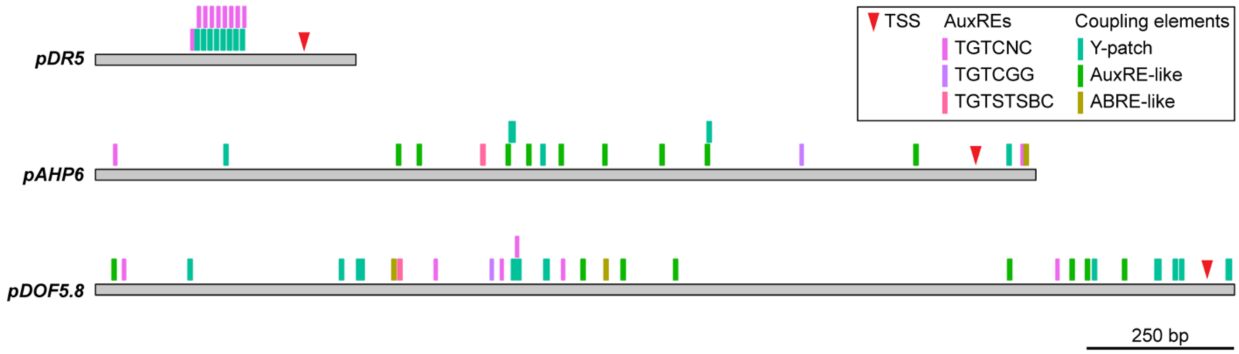

Supplementary Fig. 9. AuxREs and coupling elements in promoters of DR5, AHP6, and DOF5.8. Note the densely packed 9 AuxREs sandwiching 8 Y-patches in *pDR5*, compared to fewer AuxREs surrounded by relatively more abundant coupling elements in *pAHP6* and *pDOF5.8*.

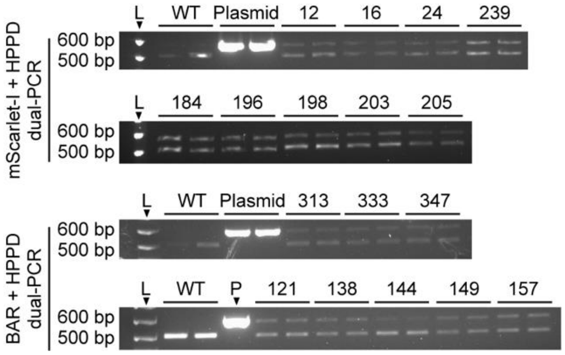

Supplementary Fig. 10. Confirmation of single insertions in reporter lines using dual-PCR of T1 plants. Band intensities of the transgenes (*mScarlet-I*, 609 bp, top, and *BAR*, 630 bp, bottom) were comparable to or slightly weaker than band intensities of the endogenous single-copy gene (*HPPD*, 520 bp), indicating single insertion. L, ladder. Source data are provided as a Source Data file.

Supplementary Table 1. Confirmation of single insertions in reporter lines using segregation ratio of antibiotic resistance in T2 seedlings. Kanamycin for mScarlet-I lines and Basta for mNG lines.

**Reporter**	**Line**	**Segregation ratio**	**Used in**
DR5::mScarlet-I-N7	239	194:51 (3.80:1)	Figure 1 and 2
DR5::mScarlet-I-N7	24	73:27 (2.70:1)	Figure 4 and 7, Supplementary Fig. 1, 2, and 8
DR5::mScarlet-I-N7	12	178:66 (2.70:1)	Supplementary Fig. 4
pAHP6::mNG-N7	313	103:40 (2.58:1)	Figure 5
pAHP6::mNG-N7	347	104:40 (2.60:1)	Supplementary Fig. 5
pAHP6::mScarlet-I-N7	184	73:27 (2.70:1)	Figure 5
pAHP6::mScarlet-I-N7	196	150:52 (2.88:1)	Supplementary Fig. 5
pDOF5.8::mNG-N7	157	180:63 (2.86:1)	Figure 6, Supplementary Fig. 7
pDOF5.8::mNG-N7	144	75:26 (2.88:1)	Supplementary Fig. 6
pDOF5.8::mScarlet-I-N7	205	71:29 (2.45:1)	Figure 6, Supplementary Fig. 7
pDOF5.8::mScarlet-I-N7	203	78:22 (3.55:1)	Supplementary Fig. 6

Supplementary Table 2. Dual PCR primer sequences.

**Name**	**Description**	**Sequence**	**Product**
oSK315	mScarlet-I-linker-N7 Fwd PCR primer	ggactcagaccgcaaagctcaa	609 bp
oSK316	mScarlet-I-linker-N7 Rev PCR primer	gctcttcacgcttgaattcggc	609 bp
oSK321	pNOS-BastaR-NOSter Fwd PCR primer	gcgcgttcaaaagtcgcctaag	630 bp
oSK322	pNOS-BastaR-NOSter Rev PCR primer	cagaaacccacgtcatgccagt	630 bp
oSK323	HPPD Fwd primer, Kihara et al. 2006	GCGCTTCCATCACATCGAGTTC	520 bp
oSK324	HPPD Rev primer, Kihara et al. 2006	AATCCAATGGGAACGACGACGC	520 bp
