# Supplementary material for: Stochastic gene expression in auxin signaling in the floral meristem of Arabidopsis thaliana
Source: Nat Commun. 2025 May 20;16:4682. doi: 10.1038/s41467-025-59943-4 (PMC12092670; doi:10.1038/s41467-025-59943-4)
Supplement: Supplementary file 2 — Description of Additional Supplementary Files [file 41467_2025_59943_MOESM2_ESM.pdf]

Description of Additional Supplementary Files

File Name: Supplementary Data 1
Description: Plasmid sequence for DR5-mScarlet-I-N7-OCSter

File Name: Supplementary Data 2
Description: Plasmid sequence for pAHP6-mNG-N7-AHP6ter

File Name: Supplementary Data 3
Description: Plasmid sequence for pAHP6-mScarlet-I-N7-AHP6ter

File Name: Supplementary Data 4
Description: Plasmid sequence for pDOF5.8-mNG-N7-DOF5.8ter

File Name: Supplementary Data 5
Description: Plasmid sequence for pDOF5.8-mScarlet-I-N7-DPF5.8ter
