# Supplementary material for: Stochastic gene expression in auxin signaling in the floral meristem of Arabidopsis thaliana
Source: Nat Commun. 2025 May 20;16:4682. doi: 10.1038/s41467-025-59943-4 (PMC12092670; doi:10.1038/s41467-025-59943-4)
Supplement: Supplementary file 8 — Reporting Summary [file 41467_2025_59943_MOESM8_ESM.pdf]

nature portfolio

Corresponding author(s): Adrienne H.K. Roeder

Last updated by author(s): Apr 30, 2025

Reporting Summary

Nature Portfolio wishes to improve the reproducibility of the work that we publish. This form provides structure for consistency and transparency in reporting. For further information on Nature Portfolio policies, see our Editorial Policies and the Editorial Policy Checklist.

Statistics

For all statistical analyses, confirm that the following items are present in the figure legend, table legend, main text, or Methods section.

n/a	Confirmed	
☐	☒	The exact sample size (*n*) for each experimental group/condition, given as a discrete number and unit of measurement
☐	☒	A statement on whether measurements were taken from distinct samples or whether the same sample was measured repeatedly
☐	☒	The statistical test(s) used AND whether they are one- or two-sided<br>*Only common tests should be described solely by name; describe more complex techniques in the Methods section.*
☒	☐	A description of all covariates tested
☐	☒	A description of any assumptions or corrections, such as tests of normality and adjustment for multiple comparisons
☐	☒	A full description of the statistical parameters including central tendency (e.g. means) or other basic estimates (e.g. regression coefficient) AND variation (e.g. standard deviation) or associated estimates of uncertainty (e.g. confidence intervals)
☐	☒	For null hypothesis testing, the test statistic (e.g. *F*, *t*, *r*) with confidence intervals, effect sizes, degrees of freedom and *P* value noted<br>*Give P values as exact values whenever suitable.*
☒	☐	For Bayesian analysis, information on the choice of priors and Markov chain Monte Carlo settings
☒	☐	For hierarchical and complex designs, identification of the appropriate level for tests and full reporting of outcomes
☒	☐	Estimates of effect sizes (e.g. Cohen's *d*, Pearson's *r*), indicating how they were calculated

Our web collection on statistics for biologists contains articles on many of the points above.

Software and code

Policy information about availability of computer code

Data collection	Confocal microscopy was done using Leica Application Suite X (LAS-X) version 4.6.1.27508.
Data analysis	Image processing was done in ImageJ (version 1.54f with Java 1.8.0_322, 64-bit), MorphoGraphX (version 2.0.1-394), and R (version 4.3.1 (2023-06-16)) run in RStudio (Version 2023.12.0+369). Figures were assembled in Adobe Illustrator (version 28.6). An RGB color profile "Image P3" was used for all the figures.

For manuscripts utilizing custom algorithms or software that are central to the research but not yet described in published literature, software must be made available to editors and reviewers. We strongly encourage code deposition in a community repository (e.g. GitHub). See the Nature Portfolio guidelines for submitting code & software for further information.

Data

Policy information about availability of data

All manuscripts must include a data availability statement. This statement should provide the following information, where applicable:

- Accession codes, unique identifiers, or web links for publicly available datasets
- A description of any restrictions on data availability
- For clinical datasets or third party data, please ensure that the statement adheres to our policy

Source data are provided with this paper.

Research involving human participants, their data, or biological material

Policy information about studies with human participants or human data. See also policy information about sex, gender (identity/presentation), and sexual orientation and race, ethnicity and racism.

Reporting on sex and gender	*Use the terms sex (biological attribute) and gender (shaped by social and cultural circumstances) carefully in order to avoid confusing both terms. Indicate if findings apply to only one sex or gender; describe whether sex and gender were considered in study design; whether sex and/or gender was determined based on self-reporting or assigned and methods used.*<br>*Provide in the source data disaggregated sex and gender data, where this information has been collected, and if consent has been obtained for sharing of individual-level data; provide overall numbers in this Reporting Summary. Please state if this information has not been collected.*<br>*Report sex- and gender-based analyses where performed, justify reasons for lack of sex- and gender-based analysis.*
Reporting on race, ethnicity, or other socially relevant groupings	*Please specify the socially constructed or socially relevant categorization variable(s) used in your manuscript and explain why they were used. Please note that such variables should not be used as proxies for other socially constructed/relevant variables (for example, race or ethnicity should not be used as a proxy for socioeconomic status).*<br>*Provide clear definitions of the relevant terms used, how they were provided (by the participants/respondents, the researchers, or third parties), and the method(s) used to classify people into the different categories (e.g. self-report, census or administrative data, social media data, etc.)*<br>*Please provide details about how you controlled for confounding variables in your analyses.*
Population characteristics	*Describe the covariate-relevant population characteristics of the human research participants (e.g. age, genotypic information, past and current diagnosis and treatment categories). If you filled out the behavioural & social sciences study design questions and have nothing to add here, write "See above."*
Recruitment	*Describe how participants were recruited. Outline any potential self-selection bias or other biases that may be present and how these are likely to impact results.*
Ethics oversight	*Identify the organization(s) that approved the study protocol.*

Note that full information on the approval of the study protocol must also be provided in the manuscript.

Field-specific reporting

Please select the one below that is the best fit for your research. If you are not sure, read the appropriate sections before making your selection.

☒ Life sciences ☐ Behavioural & social sciences ☐ Ecological, evolutionary & environmental sciences

For a reference copy of the document with all sections, see nature.com/documents/nr-reporting-summary-flat.pdf

Life sciences study design

All studies must disclose on these points even when the disclosure is negative.

Sample size	No sample size calculation was performed. Instead, the number of inflorescences to image and analyze was chosen so that clear heatmaps for the mean and variability of patterns could be generated. Usually, 6-10 inflorescences per group were enough. This gives roughly 10-20 buds for stage 1a, 1b, and 2a, and 3-10 buds for stage 2b and 2c.<br>For most of the experiments, all stage 1 and 2 buds were analyzed; for oryzalin and hydroxyurea experiments, the biggest stage 2 buds (without sepals) in each inflorescence were analyzed.
Data exclusions	Buds that were damaged during dissection were excluded from the analyses.
Replication	For the DR5 T1, DR5 dual-reporter, AHP6 dual-reporter, and DOF5.8 dual-reporter, two independent lines were imaged. One of them was in the main figures; the other was in the supplemental figures. Most conclusions were consistent between the two lines. For the DOF5.8 dual marker, intrinsic noise was much higher in the second line (Supplementary Fig. 6d) than the first line (Fig. 6e).
Randomization	For oryzalin, hydroxyurea, and 2,4-D treatments, dissected inflorescences were randomly assigned to either mock or drug treatment groups. Other experiments in this study did not involve group assignment and thus did not involve randomization.
Blinding	The investigators were not blinded to group labels. Blinding was not possible because the groups labels were written explicitly on tissue culture plates and in file names during the process of data acquisition and analysis.

Reporting for specific materials, systems and methods

We require information from authors about some types of materials, experimental systems and methods used in many studies. Here, indicate whether each material, system or method listed is relevant to your study. If you are not sure if a list item applies to your research, read the appropriate section before selecting a response.

Materials & experimental systems

n/a	Involved in the study
☒	☐ Antibodies
☒	☐ Eukaryotic cell lines
☒	☐ Palaeontology and archaeology
☒	☐ Animals and other organisms
☒	☐ Clinical data
☒	☐ Dual use research of concern
☐	☒ Plants

Methods

n/a	Involved in the study
☒	☐ ChIP-seq
☒	☐ Flow cytometry
☒	☐ MRI-based neuroimaging

Plants

Seed stocks	DR5::3xVENUS-N7 stock was a gift from the Vernoux Lab. R2D2 (Col-Utr background) was a gift from the Weijers Lab.
Novel plant genotypes	All Arabidopsis (Arabidopsis thaliana, RRID:NCBITaxon_3702) plants were in Col-0 background (WT). For single reporters of DR5::mScarlet-I-N7, pAHP6::mScarlet-I-N7, and pDOF5.8::mScarlet-I-N7, plants already carrying the pATML1::H2B-TFP construct were transformed with the respective mScarlet-I constructs using the floral dip method, and T2 plants were imaged. For the DR5 dual reporter, plants carrying both pATML1::H2B-TFP and DR5::mScarlet-I-N7 were crossed with DR5::3xVENUS-N7 plants, and F1 plants were imaged. For pAHP6 and pDOF5.8 dual reporters, Col-0 plants were transformed with the respective mNG constructs. T1 plants were crossed with plants carrying both pATML1::H2B-TFP and the respective mScarlet markers, and F1 plants were imaged. For R2D2, the original line in Columbia-Utrecht background was backcrossed with Col-0 twice before using.
Authentication	All T1 transformants were screened using dual-PCR for single-copy insertion lines. These single-insertion lines were confirmed by counting segregation ratio of antibiotic resistance in T2. Transformant plants were imaged to ensure the correct expression of the transgenes.
